# DL-QC-fNIRS: a deep learning tool for automated quality control in functional near-infrared spectroscopy signals

**DOI:** 10.1117/1.NPh.13.1.015001

**Published:** 2025-12-29

**Authors:** Sabino Guglielmini, Zhuofei Chen, Martin Wolf

**Affiliations:** a University Hospital Zurich, University of Zurich, Biomedical Optics Research Laboratory, Department of Neonatology, Zurich, Switzerland; b ETH Zurich, Department of Information Technology and Electrical Engineering (D-ITET), Zurich, Switzerland

**Keywords:** functional near-infrared spectroscopy, fNIRS, signal quality, deep learning, wavelet transform, fNIRS preprocessing

## Abstract

**Significance:**

In functional near-infrared spectroscopy (fNIRS) research, ensuring signal quality is a critical preprocessing step. However, traditional index-based metrics such as the coefficient of variation (CV) and scalp coupling index (SCI) rely on arbitrary thresholds and often misclassify channels.

**Aim:**

We present DL-QC-fNIRS, a deep learning framework for the automated, channel-wise assessment of signal quality.

**Approach:**

Our method involves generating continuous wavelet transform scalograms of oxyhemoglobin signals and employing subject-specific cardiac frequency extraction to improve physiological specificity. These inputs are then classified using convolutional neural networks (CNNs). We benchmarked four CNN architectures (GoogLeNet, ResNet-50, SqueezeNet, and EfficientNet-B0) on two independent datasets and one combined heterogeneous dataset.

**Results:**

GoogLeNet achieved the highest accuracy (>93%) on the combined dataset, demonstrating strong sensitivity and specificity across test sets. Compared with CV and SCI, DL-QC-fNIRS yielded markedly higher F1-scores and a more favorable balance between sensitivity and specificity.

**Conclusions:**

DL-QC-fNIRS is provided as an open-source MATLAB-based graphical interface, enabling accessible and standardized integration into fNIRS workflows. These findings highlight DL-QC-fNIRS as a scalable, expert-level tool for improving the reliability and reproducibility of optical neuroimaging data.

## 1 Introduction

Functional near-infrared spectroscopy (fNIRS) is a popular neuroimaging modality that enables noninvasive monitoring of cerebral hemodynamics at a relatively low cost and with high portability.^1^^–^^3^ However, the reliability of fNIRS data is dependent on signal quality, i.e., the stability and cleanliness of the optical measurement. When the optode is well coupled to the scalp, the signal is stable, and physiological fluctuations such as the cardiac pulse are clearly detectable. Poor optode-scalp coupling, subject motion, or physiological interference may generate low-quality channels that contaminate the data and compromise the validity of experimental findings.^4^^–^^7^ Previous studies have shown that failing to identify and remove such channels may bias group-level results, yet only a minority of published reports explicitly describe formal low-quality channel detection procedures.^8^^–^^10^ In many cases, researchers continue to rely on ad hoc or subjective assessments, such as visual inspection of time series, which are difficult to standardize and reproduce.^11^ This highlights the need for objective, automated, and widely applicable methods of quality control in fNIRS research.^12^^,^^13^

Ensuring data quality is a critical preprocessing step in fNIRS studies. Several signal quality metrics have been suggested as a way of addressing this issue. Simple approaches, such as the coefficient of variation (CV) or the signal-to-noise ratio, provide convenient heuristics, but they require threshold values that are often chosen arbitrarily and that vary across studies and devices.^4^^,^^5^ More physiologically informed measures, such as the scalp coupling index (SCI) or the detection of cardiac pulsations, offer a stronger basis by directly evaluating whether the heartbeat is present in the optical signal.^14^^–^^16^ However, these approaches may still be affected by transient artifacts, or they may exclude channels that contain some physiological information but fall below a strict cut-off point.^9^^,^^12^ More recent multifeature indices attempt to integrate several criteria into a composite score, thereby improving accuracy but increasing complexity and requiring dataset-specific calibration.^9^

In general, most existing metrics have two major limitations: (i) they depend on fixed, user-defined thresholds, and (ii) they rely on static or aggregate assessments that overlook temporal variations in quality. In practice, the quality of a channel is rarely stable throughout an entire recording. For instance, a channel that initially exhibits strong pulsations may deteriorate later due to optode displacement, whereas a brief motion artifact may temporarily reduce the quality index without rendering the channel unusable overall. Therefore, an ideal method should be sensitive to time-varying behavior while retaining robustness against short-lived disturbances. Furthermore, manually inspecting time and frequency representations, such as wavelet spectra, reveals patterns that are meaningful from a physiological perspective, including the presence and continuity of cardiac, respiratory, or low-frequency oscillations. However, this process is labor-intensive and impractical for large datasets.^17^^,^^18^ These challenges suggest that a more flexible, deep learning (DL)-based framework is needed, one that recognizes the complex patterns that distinguish between high- and low-quality fNIRS channels.^5^^,^^19^

In this study, the aim is to present DL-QC-fNIRS, a DL framework designed for the automated evaluation of fNIRS channel quality.

## 2 Materials and Methods

### 2.1 Dataset and Annotation

The data for this study were obtained from two independent resting-state fNIRS datasets, which were collected under distinct experimental protocols. Written informed consent was obtained from all participants, and both studies were approved by the Ethics Committee of the County of Zurich.

**Fig. 1:**
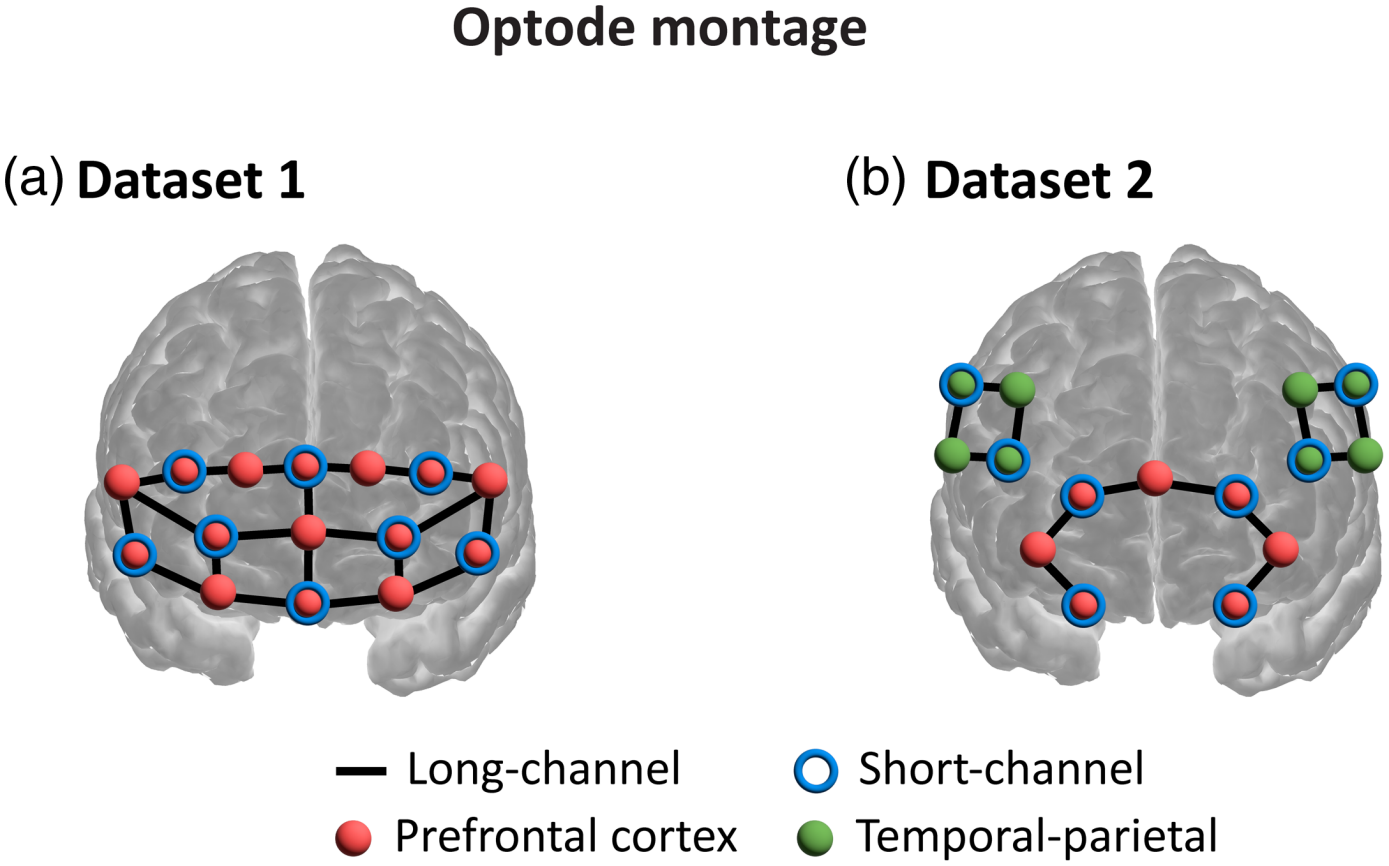
Optode configurations for the two datasets. (a) Montage used in Dataset 1: 20 long-separation channels (30 mm) and eight frontal short-separation channels (8 mm) acquired with the NIRSport 1 system. (b) Montage used in Dataset 2: 14 long-separation channels (six prefrontal, four left temporo-parietal, and four right temporo-parietal) and eight short-separation channels acquired with the NIRSport 2 system.

Dataset 1 consisted of 40 recordings from healthy adults, acquired using a NIRSport 1 system (NIRx Medical Technologies GmbH). The montage included 20 long-separation channels (30 mm) and eight short-separation channels (8 mm) positioned over the frontal region [see Fig. 1(a)]. Each recording lasted 1780 s, during which participants were instructed to remain still and relaxed. The system employed eight single-tip LED sources and eight silicon photodiode detectors, sampling at 7.812 Hz at 760 and 850 nm. Each channel was divided into five 356-s segments, resulting in 5600 segments in total (40 subjects × 28 channels × 5). Annotation of the segments was performed using our custom graphical user interface (GUI) (see Sec. 4) by a single expert annotator, yielding 3347 low-quality (59.8%) and 2253 high-quality (40.2%) samples.

Dataset 2 comprised 92 recordings from a previous hyperscanning study involving healthy adults, which were acquired using an NIRSport 2 system (NIRx Medical Technologies GmbH, Berlin, Germany). The montage comprised 14 long-separation channels (six prefrontal, four left temporo-parietal, and four right temporo-parietal) and eight short-separation channels [see Fig. 1(b)]. Participants underwent 2312 s of eyes-closed rest while minimizing movement. The NIRSport 2 system used eight dual-tip LED sources and eight avalanche photodiode detectors, with acquisition at 760 and 850 nm and a sampling rate of 8.719 Hz. Each channel was divided into five 462.5-s segments, yielding 5060 samples. The annotation produced 3227 high-quality (63.8%) and 1833 low-quality (36.2%) segments.

All segments from both datasets were annotated using our custom GUI (see Sec. 4) based on visual inspection of the continuous wavelet transform (CWT) scalograms. A segment was labeled high-quality when a continuous and stable cardiac-band oscillation was clearly visible across the entire time window. A segment was labeled low-quality when the cardiac pulsation was absent, intermittent, or masked by artifacts, including motion-related broadband bursts or detector saturation. Minor motion artifacts occasionally occurred; segments in which motion suppressed the cardiac component were labeled as low-quality according to these criteria.

We also combined the two datasets by concatenating all samples and their labels. The resulting combined dataset contained 10,660 channel segments, with a nearly even distribution of 5480 high-quality (51.4%) and 5180 low-quality (48.6%) samples. The intention behind the fusion was to improve the generalizability of the model by exposing the network to diverse acquisition systems, montages, and signal quality levels during training.

### 2.2 Preprocessing

First, raw light intensity signals were converted to optical density.^20^ Then, concentration changes in oxyhemoglobin ($\left[O_{2}\mathrm{Hb}\right]$) were computed using the modified Beer–Lambert law with a differential pathlength factor of 6.^21^^–^^24^ We focused on oxyhemoglobin signals to generate CWT representations because, compared with deoxyhemoglobin ([HHb]) signals, they provide higher contrast and a better signal-to-noise ratio during neural activation.^1^ This choice results in stable and physiologically specific features in the time-frequency domain for subsequent classification.^25^ Filtering and transformation were performed on $\left[O_{2}\mathrm{Hb}\right]$ rather than optical density, as previous studies have shown no significant differences between the two approaches in terms of the final statistical outcomes.^26^

To extract time-frequency features related to cardiac pulsation, we employed CWT and visualized the resulting scalograms.^27^^,^^28^ Conventional approaches typically rely on band-pass filtering within the fixed cardiac range of 0.5 to 2.5 Hz (30 to 150 beats per minute, bpm) to isolate the cardiac signal from cerebral hemodynamics and other physiological noise.^13^ However, this broad frequency window does not account for inter-individual variability in heart rate, which may depend on age, health status, and experimental conditions. Moreover, the wide bandwidth may inadvertently capture noise peaks in adjacent frequency ranges. To address these limitations, we implemented a spectral fitting algorithm that automatically identifies an individualized cardiac frequency band. By adapting the bandwidth to each participant, this method enables more precise isolation of cardiac features and minimizes contamination from nonphysiological noise.

### 2.3 Spectral Fitting Algorithm

#### 2.3.1 Cardiac peak detection

The algorithm begins by estimating the power spectral density (PSD) of each $\left[O_{2}\mathrm{Hb}\right]$ signal using Welch’s method with a Hamming window and 50% overlap.^29^^,^^30^ To reduce noise while preserving the morphology of spectral peaks, the PSD is then smoothed using a third-order Savitzky–Golay polynomial filter.^31^^–^^33^

As fNIRS spectra exhibit a strong aperiodic 1/f-like background, accurately detecting the cardiac peak requires its removal.^27^^,^^34^ Following approaches from spectral parameterization in neuroscience, a third-order polynomial is fitted to the log-transformed power spectrum (dB scale) to model this aperiodic component.^35^ Subtracting this fit yields a flattened spectrum in which periodic peaks are more clearly distinguishable.^36^^–^^38^

**Fig. 2:**
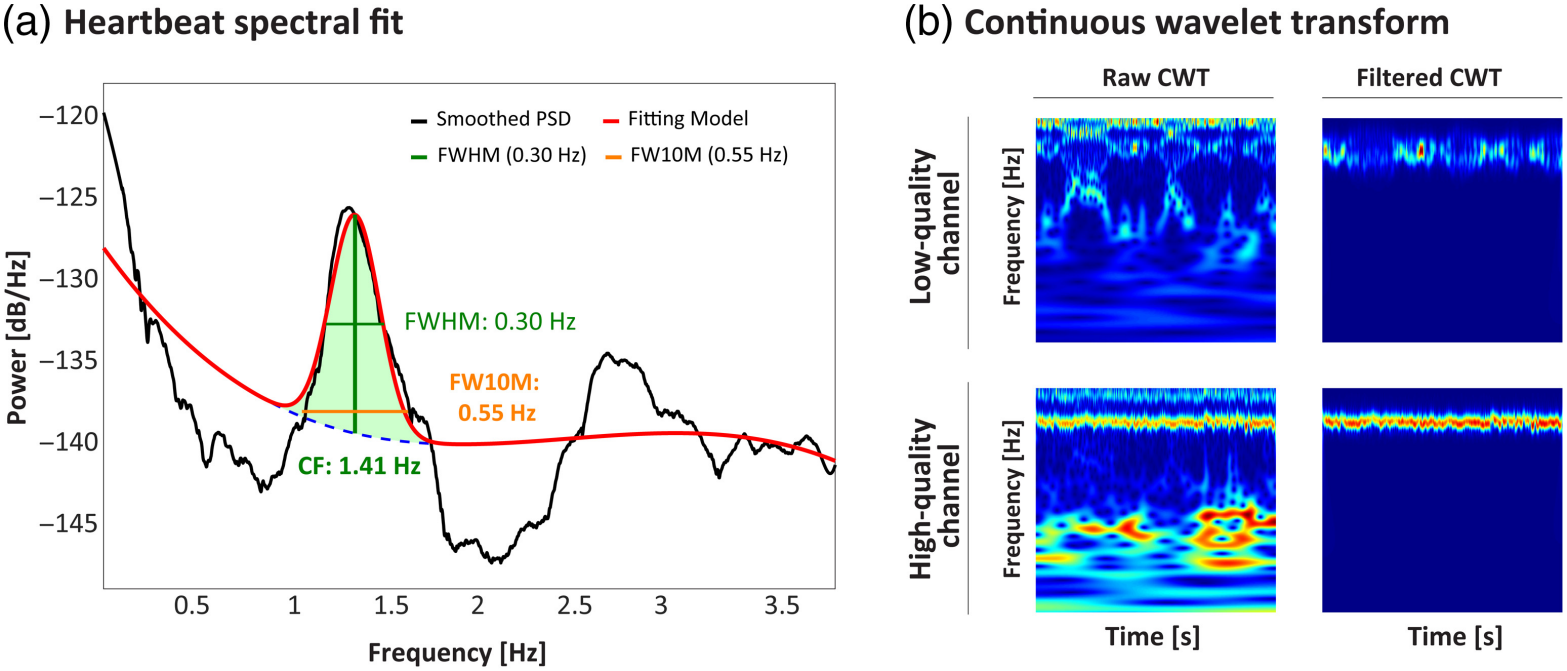
Spectral fitting and time–frequency representation of fNIRS signals. (a) Example of the heartbeat spectral fitting process. The PSD of the $\left[O_{2}\mathrm{Hb}\right]$ signal is estimated using Welch’s method, smoothed with a Savitzky–Golay filter, and flattened by removing the aperiodic 1/f background. A Gaussian fit is applied to the most prominent peak to precisely estimate its center frequency (CF) and bandwidth, defined as the full width at 10% of the maximum (FW10M), which defines the subject-specific cardiac band. (b) Continuous wavelet transform (CWT) scalograms for a high-qualityand a low-quality channel. For each, the left panel shows the full CWT spectrogram computed using the Morlet wavelet, while the right panel shows the filtered and frequency-aligned CWT scalogram used as input to the CNN. The cardiac band identified in the previous step is used to filter and normalize each scalogram, standardizing feature location across subjects and improving classification performance.

Peak detection is then performed across the entire frequency range to account for variability between populations (e.g., elevated heart rates in infants).^39^ To enhance robustness, only peaks with a minimum prominence of >0.5  dB are retained.^35^ For each detected peak, we fitted a Gaussian function to estimate its center frequency (CF), power, and full width at half maximum (FWHM), yielding precise, individualized measures of cardiac spectral features.^40^ Figure 2(a) illustrates the result of this spectral flattening and peak detection process.

To represent the bandwidth, we used the full width at 10% of the maximum (FW10M). This metric was chosen over the more conventional FWHM as it provides a wider, more conservative estimate that better captures the full extent of the cardiac spectral peak, enhancing robustness against variations in peak shape.^41^^,^^42^

To ensure cardiac bands are defined using the most reliable data, we applied a channel preselection procedure using the QT-NIRS toolbox, the most commonly used toolkit for fNIRS channel pruning in recent pipelines.^9^^,^^13^ QT–NIRS evaluates channel quality using metrics like a composite quality index (e.g., combined SCI and PSD measures).^43^ In our iterative procedure, we first select channels exceeding stringent thresholds (composite quality index >0.9, SCI>0.8, and cardiac PSD peak>0.1). If no channels meet these criteria, we gradually relax the composite quality index (CQI) threshold until at least one high-quality channel remains for analysis. The spectral fitting algorithm is then applied only to this subset. To derive subject-level estimates, we calculated the median CF and FW10M values across all best channels, reducing the influence of outliers. The individualized cardiac band is then defined as ${\mathrm{CF}}_{\left(\text{median}\right)}\pm {\mathrm{FW}10M}_{\left(\text{median}\right)}/2$, providing a narrow, robust frequency range for subsequent feature extraction.

#### 2.3.2 CWT-based time-frequency representation and standardization

The CWT was used to generate two-dimensional time–frequency representations (scalograms) from the one-dimensional fNIRS signals, enabling localized analysis of oscillatory activity across time and frequency.^44^^,^^45^ First, each segment was band-pass filtered within the subject-specific dynamic cardiac band defined in Sec. 2.3.1 to isolate pulsatile activity. The CWT was then computed using the Morlet wavelet, which is well-suited to modeling oscillatory physiological signals.^46^ The magnitude of the resulting complex-valued CWT formed the scalogram. To ensure compatibility with the convolutional neural network (CNN), the scalograms were normalized across all segments within each channel to ensure consistent contrast and then converted into RGB images using the jet colormap.^47^ Figure 2(b) illustrates representative scalograms highlighting the visual differences between high-quality segments (continuous cardiac-band structure) and low-quality segments (absent or fragmented pulsation or artefactual broadband activity).

To improve classification accuracy and reduce variability arising from inter-subject differences in heart rate, a scalogram standardization procedure was implemented. As the location of the cardiac band varies between subjects, relevant features appear at different vertical positions in raw scalograms. To align these features, the subject-specific cardiac band was shifted to a fixed reference row (row 30) by cropping and padding along the frequency axis. The time axis was then rescaled to a uniform width using bilinear interpolation. The resulting 224×224-pixel RGB images contained the cardiac features in a consistent spatial location, enabling the CNN to identify physiologically meaningful patterns regardless of the heart rate. This normalization step substantially enhanced both classification accuracy and model generalization.

### 2.4 Convolutional Neural Network Architectures

Deep CNNs with transfer learning were used to classify fNIRS scalograms.^48^^–^^50^ Four representative pre-trained architectures were evaluated to compare different design strategies in terms of computational efficiency and performance. These were SqueezeNet, which uses fire modules to reduce parameters^51^; GoogLeNet, which introduced inception modules for multiscale feature extraction^52^; ResNet-50, which has 50 layers and uses skip connections to enable the stable training of deep models^53^; and EfficientNet-B0, which uses compound scaling to adjust depth, width, and resolution.^54^

To adapt each model to the binary classification task (high-quality versus low-quality channels), the final feature extraction and classification layers were replaced. To accelerate convergence while retaining the pretrained feature hierarchies, the learning rate factors for the new layers were increased tenfold.^55^^,^^56^ All experiments were implemented in MATLAB R2023b (deep learning toolbox). Training was performed on a workstation equipped with a 12th Gen Intel Core i7-12700H CPU (2.30 GHz), 16 GB of RAM, and a 64-bit Windows operating system. Model training was accelerated using an NVIDIA GeForce GTX 1080 Ti GPU (11 GB VRAM) with the NVIDIA CUDA v12.5 library.

To prevent data leakage and address inter-subject variability, we employed a subject-wise stratified protocol, ensuring that all samples from each subject were assigned exclusively to either the training or the test set.^57^^,^^58^

Each dataset was first divided at the subject level into a development set (90% of subjects) and an independent hold-out test set (10% of subjects). A combined dataset was then created by merging the corresponding development and test subsets from both sources. Within each development set, we performed subject-wise stratified 10-fold cross-validation to optimize model architecture and hyperparameters while ensuring that data from the same subject were never split across folds.^59^^,^^60^ To address the unequal class distribution, class weights were incorporated into the loss function.^61^ Regularization strategies included L2 weight decay ($1\times 10^{-4}$), on-the-fly data augmentation (random translation, scaling, and cropping), and early stopping if validation accuracy did not improve for 15 consecutive epochs.^62^^,^^63^ All models were trained using the Adam optimizer with an initial learning rate of $1\times 10^{-4}$, a batch size of 32, and a maximum of 50 epochs.^64^ Identical hyperparameters were used across all architectures and datasets to ensure comparability. After cross-validation, the best-performing model was retrained on the entire 90% development set and evaluated on the independent 10% hold-out test set.

### 2.5 Performance Evaluation Metrics

Model performance for binary classification of fNIRS signal quality was evaluated using standard confusion matrix-based metrics.^65^ In our setup, the detection of a low-quality signal was a positive, and the detection of a high-quality signal was a negative. We computed the following measures:

• Accuracy = (TP + TN)/(TP + TN + FP + FN).
• Precision = TP/(TP + FP).
• Recall (Sensitivity) = TP/(TP + FN).
• Specificity = TN/(TN + FP).
• F1-score = 2 × (Precision × Recall)/(Precision + Recall).

where TP, TN, FP, and FN represent true positives, true negatives, false positives, and false negatives, respectively. Results are reported as mean ± standard deviation across ten cross-validation folds and on an independent test set.

For benchmarking purposes, our method was compared against two widely used index-based indices: (i) the CV and (ii) the SCI. CV quantifies variability in raw light intensity as the ratio of the standard deviation to the mean, expressed as a percentage.^4^^,^^66^ A higher CV indicates greater signal fluctuation and thus a lower signal-to-noise ratio. Although simple to compute, its primary limitations are its reliance on subjectively defined thresholds for channel rejection and its sensitivity to motion artifacts. For our comparison, we adopted a commonly used threshold, classifying signals with a CV>15% as low-quality.^67^ The SCI evaluates signal quality by computing the normalized zero-lag cross-correlation between two wavelengths of a channel to estimate the strength of cardiac pulsations.^13^^,^^68^ We used the default quality threshold of 0.75 recommended in the original study.^13^ Although the SCI is physiologically informed, it depends on subjective parameter tuning (e.g., window length and cutoff values) and applies a fixed cardiac band (0.5 to 2.5 Hz). This makes it vulnerable to contamination from motion artifacts within this wide range.^69^

### 2.6 Statistical Analysis

To compare the performance of the deep-learning architectures during cross-validation, we used the nonparametric Friedman test to both accuracy and F1-score across the ten folds.^70^ When significant differences were indicated, pairwise Wilcoxon signed-rank tests with the Benjamini-Hochberg false discovery rate (FDR) correction were applied for posthoc analysis.^71^ Cliff’s δ was computed as a nonparametric effect size for each pairwise comparison.^72^^,^^73^

To compare the final DL-QC-fNIRS model with the benchmark methods (CV and SCI) on the independent test sets, we used McNemar’s test with continuity correction, which evaluates whether two paired classifiers differ significantly in their misclassification patterns.^74^

All statistical analyses were conducted in R (version 4.4.3) within RStudio (2025.09.1).^75^^,^^76^ Statistical significance was set at p<0.05.

## 3 Results

We first report the cross-validation results for the four candidate CNN architectures, which guided the selection of the final model. We then evaluate the chosen model on independent test sets and benchmark its performance against traditional index-based metrics.

### 3.1 Cross-validation Performance

All four CNN architectures were evaluated on the development sets of datasets 1 and 2, as well as the combined dataset, using a 10-fold, subject-wise cross-validation protocol. This analysis provided insight into how performance varies with data quality and heterogeneity and informed the selection of the optimal architecture. Detailed results are provided in Tables 1–3.

**Table 1:** Cross-validation performance of four CNN architectures on dataset 1 (10-fold subject-wise validation). Metrics are reported as mean ± standard deviation. Low-quality signals were defined as the positive class. GoogLeNet achieved the highest overall accuracy and F1-score, whereas EfficientNet-B0 required substantially longer training time despite comparable predictive performance.

Model	Accuracy (%)	Precision (%)	Recall (%)	F1-score (%)	Specificity (%)	Time (min)
SqueezeNet	89.16 ± 1.06	90.72 ± 2.04	90.55 ± 2.48	90.59 ± 0.91	87.08 ± 3.58	3.23
GoogLeNet	89.20 ± 1.17	91.42 ± 1.35	89.77 ± 1.74	90.57 ± 0.99	88.30 ± 2.31	4.31
ResNet-50	88.55 ± 1.10	89.94 ± 1.14	90.29 ± 1.38	90.11 ± 0.91	85.80 ± 1.97	4.42
EfficientNet-B0	88.81 ± 0.88	90.41 ± 1.42	90.23 ± 1.83	90.30 ± 0.75	86.56 ± 2.51	23.71

**Table 2:** Cross-validation performance of four CNN architectures on dataset 2 (10-fold subject-wise validation). Metrics are reported as mean ± standard deviation. GoogLeNet achieved the highest accuracy and F1-score, confirming its superior generalization on higher-quality signals.

Model	Accuracy (%)	Precision (%)	Recall (%)	F1-score (%)	Specificity (%)	Time (min)
SqueezeNet	90.58 ± 1.07	88.76 ± 3.47	85.72 ± 3.38	87.11 ± 1.40	93.58 ± 2.48	2.57
GoogLeNet	91.00 ± 0.89	88.46 ± 3.05	87.35 ± 2.38	87.83 ± 1.01	93.26 ± 2.26	8.34
ResNet-50	89.63 ± 1.62	85.39 ± 3.01	87.29 ± 3.36	86.27 ± 2.09	91.07 ± 2.34	16.06
EfficientNet-B0	90.16 ± 0.79	86.24 ± 2.98	87.87 ± 4.19	86.92 ± 1.20	91.57 ± 2.54	43.59

**Table 3:** Cross-validation performance of four CNN architectures on the combined dataset (10-fold subject-wise validation). Metrics are reported as mean ± standard deviation. GoogLeNet achieved the highest accuracy and F1-score, consolidating its selection as the final model.

Model	Accuracy (%)	Precision (%)	Recall (%)	F1-score (%)	Specificity (%)	Time (min)
SqueezeNet	92.40 ± 0.96	92.82 ± 1.79	91.20 ± 2.04	91.98 ± 0.98	93.81 ± 2.12	12.48
GoogLeNet	93.10 ± 1.09	93.39 ± 2.21	92.07 ± 1.31	92.71 ± 1.00	94.34 ± 2.50	26.09
ResNet-50	92.50 ± 1.18	91.93 ± 1.80	92.51 ± 1.31	92.21 ± 1.12	92.69 ± 2.09	16.12
EfficientNet-B0	92.23 ± 1.32	91.68 ± 2.48	92.26 ± 1.29	91.95 ± 1.19	92.40 ± 2.86	83.66

#### 3.1.1 Performance on the dataset 1

Dataset 1 represented the most challenging condition as it contained the highest proportion of low-quality signals. Across models, the mean test accuracies from the 10-fold cross-validation were tightly clustered between 88.55% and 89.20% (see Table 1). GoogLeNet achieved the highest accuracy (89.20%±1.17%) and F1-score (90.57%±0.99%). SqueezeNet, ResNet-50, and EfficientNet-B0 produced similar results, although with slightly lower specificity. Training times varied considerably, with EfficientNet-B0 requiring substantially more computation time despite achieving a similar level of predictive accuracy. Statistical testing across the 10 folds showed no evidence of overall differences among the four deep-learning architectures (p>0.05).

#### 3.1.2 Performance on the dataset 2

When evaluated using the higher-quality signals from dataset 2, all architectures showed improved performance compared to dataset 1 (see Table 2). Accuracies ranged from 89.63% to 91.00%. GoogLeNet achieved the best overall results once again, with the highest accuracy (91.00%±0.89%) and F1-score (87.83%±1.01%), thus confirming its robustness across dataset. The Friedman test indicated a significant overall effect on accuracy ($\chi^{2}=9.12$, p=0.028), but posthoc Wilcoxon tests with FDR correction showed no significant pairwise differences (all p≈0.18).

#### 3.1.3 Performance on the combined dataset

All architectures performed best on the combined dataset, confirming the advantage of training on a larger, more heterogeneous dataset (see Table 3). GoogLeNet once again emerged as the top performer, achieving a peak accuracy of 93.10%±1.09% and the highest F1-score of 92.71%±1.00%. ResNet-50 and EfficientNet-B0 also performed strongly, whereas SqueezeNet provided a reliable, albeit less accurate, baseline. A clear trade-off between accuracy and efficiency was observed: SqueezeNet was the fastest to train, whereas EfficientNet-B0 incurred the heaviest computational cost. The Friedman tests showed no significant overall differences among architectures for either accuracy ($\chi^{2}=7.32$, p=0.062), indicating statistically comparable performance across models. Accordingly, GoogLeNet was selected for independent testing based on its consistently highest mean performance and computational efficiency, rather than on statistically significant pairwise differences.

### 3.2 Final Evaluation and Comparison with Existing Methods

Following cross-validation, the selected GoogLeNet architecture was retrained on each dataset and evaluated on the corresponding independent test set. Its performance was benchmarked against two widely used index-based indices: the CV and the SCI.

#### 3.2.1 Performance on the dataset 1

Table 4 summarizes the comparative performance on the dataset 1 test set. The proposed DL model achieved an accuracy of 89.11% and an F1-score of 92.46%, reflecting a favorable trade-off between sensitivity and specificity. By contrast, the CV method acted as an overly conservative filter, achieving near-perfect specificity (99.33%) but extremely poor recall (23.66%). This indicates that it preserved most high-quality signals but failed to identify the majority of low-quality ones. The SCI method yielded more moderate recall but had very low specificity (47.8%), misclassifying a large proportion of high-quality channels as low-quality. Overall, our model demonstrated a more favorable sensitivity–specificity profile, successfully detecting low-quality channels while retaining high-quality data reliably. To formally validate these observations, we performed McNemar’s tests on the independent test-set predictions. DL-QC-fNIRS showed statistically significant improvements over both SCI ($\chi^{2}=48.79$, p<0.001) and CV ($\chi^{2}=352.00$, p<0.001), confirming that the observed performance gains are unlikely to be due to chance.

**Table 4:** Independent test set performance on dataset 1. DL–QC–fNIRS (with GoogLeNet) achieved the highest balanced accuracy and F1-score, outperforming the CV and SCI methods, which suffered from extreme recall–specificity trade-offs.

Method	Accuracy (%)	Precision (%)	Recall (%)	F1-score (%)	Specificity (%)
CV	63.93	96.88	23.66	38.04	99.33
SCI	82.32	82.26	96.01	88.61	47.80
DL-QC-fNIRS (with GoogLeNet)	89.11	91.67	93.27	92.46	78.62

**Fig. 3:**
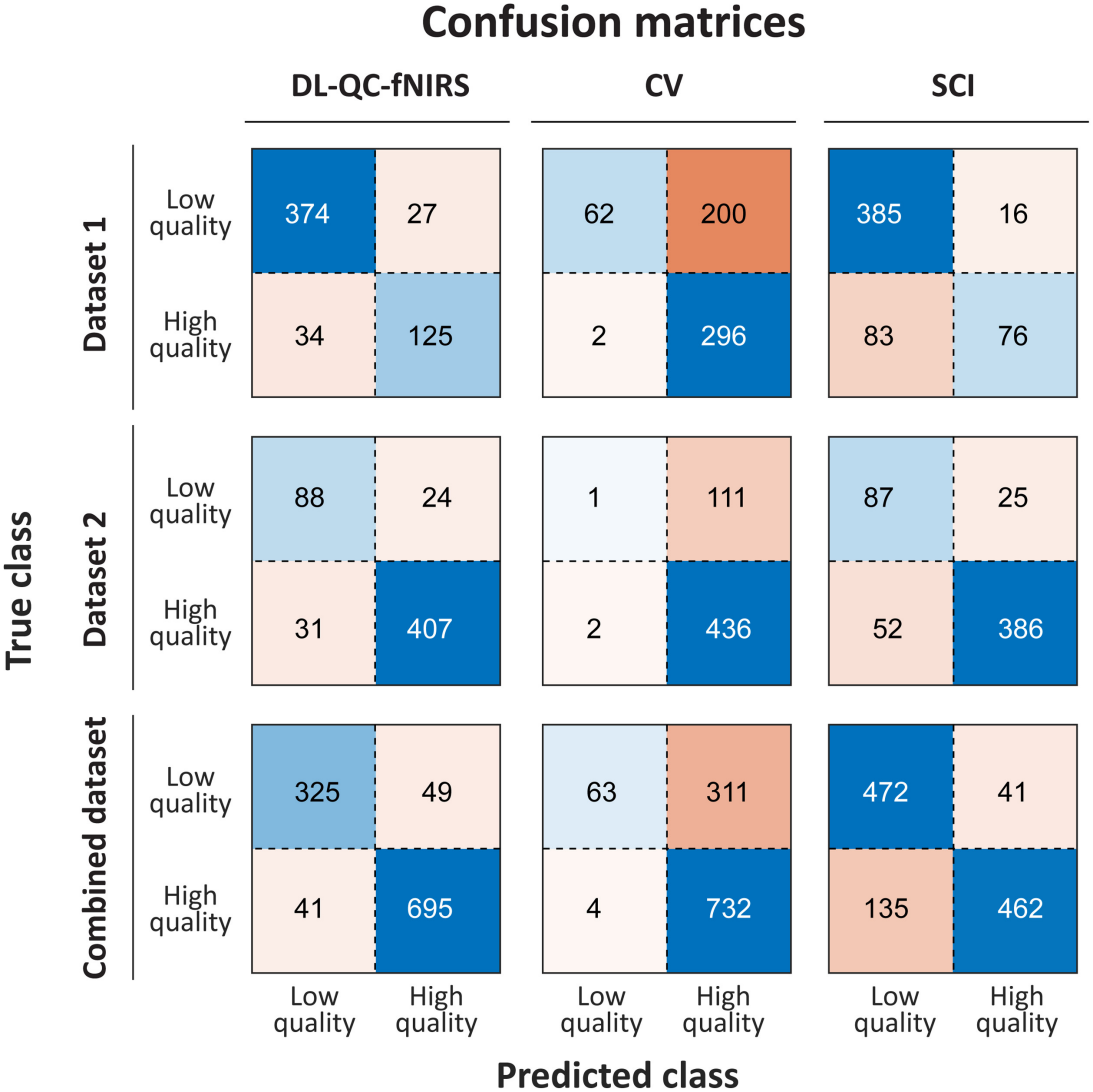
Confusion matrices comparing the classification performance of the three quality assessment methods: DL-QC-fNIRS, coefficient of variation (CV), and scalp coupling index (SCI). The top panel shows results on dataset 1, the middle panel on dataset 2, and the bottom panel on the combined dataset. The proposed method consistently achieves a more favorable sensitivity–specificity trade-off, whereas CV and SCI suffer from skewed performance patterns.

The confusion matrices in Fig. 3 further illustrate these differences. For dataset 1 (top panel), the CV method yields very few false positives but fails to correctly identify most low-quality signals, consistent with its extremely low recall. SCI achieves higher recall but at the expense of many false positives, reflected in its poor specificity. By contrast, DL-QC-fNIRS provides a more consistent classification performance across both classes, with substantially fewer misclassifications in both classes.

#### 3.2.2 Performance on the dataset 2

On the dataset 2, where low-quality signals represent a small minority, the proposed model once again demonstrated robust performance across both classes (see Table 5). It achieved an accuracy of 90.00% and an F1-score of 76.19% for the minority low-quality class, whereas maintaining excellent specificity (92.92%) and retaining most high-quality signals. By contrast, the CV method failed to detect low-quality channels effectively (recall = 0.89% and F1-score = 1.74%), despite achieving near-perfect specificity. The SCI method performed better but still yielded lower F1-scores and specificity than our approach.

**Table 5:** Independent test set performance on dataset 2. The proposed model achieved the best overall sensitivity–specificity performance, outperforming CV and SCI, which suffered from extreme recall–specificity trade-offs.

Method	Accuracy (%)	Precision (%)	Recall (%)	F1-score (%)	Specificity (%)
CV	79.45	33.33	0.89	1.74	99.54
SCI	86.00	62.59	77.68	69.32	88.13
DL-QC-fNIRS (with GoogLeNet)	90.00	73.95	78.57	76.19	92.92

The confusion matrices in Fig. 3 (middle panel) confirm these trends: CV acts as an overly conservative filter, misclassifying nearly all low-quality channels as high-quality; meanwhile, SCI shows improved sensitivity, albeit at the cost of misclassifying many high-quality signals. DL-QC-fNIRS achieves the best trade-off, with relatively few misclassifications in either class. McNemar’s tests supported these findings. The difference between DL-QC-fNIRS and CV was statistically significant ($\chi^{2}=143.06$, p<0.001), whereas the comparison with SCI was not ($\chi^{2}=1.30$, p=0.254), indicating that although the DL model achieved higher metrics, their error patterns on this dataset were not significantly different.

#### 3.2.3 Performance on the combined dataset

Evaluation on the combined test dataset, which best reflects real-world heterogeneity, confirmed the superior generalization of our proposed method (see Table 6). GoogLeNet achieved the highest accuracy (91.89%), as well as a strong F1-score (87.84%) for the low-quality class and excellent specificity (94.43%) for the high-quality class. This suggests that the model effectively detects poor-quality signals and reliably preserves high-quality ones, achieving a strong sensitivity–specificity trade-off that the benchmark methods failed to attain.

**Table 6:** Independent test set performance on the combined dataset. The proposed model achieved the best overall sensitivity–specificity performance, combining high sensitivity for low-quality signals with strong specificity for high-quality signals.

Method	Accuracy (%)	Precision (low-quality) (%)	Recall (low-quality) (%)	F1-score (low-quality) (%)	Specificity (%)
CV	71.62	94.03	16.84	28.57	99.46
SCI	84.14	77.76	92.00	84.26	77.37
DL-QC-fNIRS (with GoogLeNet)	91.89	88.80	86.90	87.84	94.43

By contrast, CV acted as an overly conservative filter, achieving near-perfect specificity (99.46%) but extremely poor recall (16.84%), resulting in a low F1-score of 28.57%. SCI offered better sensitivity (92.00%), albeit at the expense of specificity (77.37%), resulting in a substantial number of false positives.

The confusion matrices in Fig. 3 (bottom panel) reinforce these findings: CV misclassifies most poor-quality channels as high-quality, whereas SCI misclassifies many high-quality channels as poor quality. By contrast, DL-QC-fNIRS provides the most consistent classification across both classes. On the combined dataset, McNemar’s tests showed that DL-QC-fNIRS significantly outperformed both SCI ($\chi^{2}=18.99$, p<0.001) and CV ($\chi^{2}=496.03$, p<0.001), demonstrating consistent statistical robustness across heterogeneous recording conditions.

## 4 Software Implementation

### 4.1 Software Overview

All preprocessing procedures and the DL-based quality assessment framework using CWT scalograms have been integrated into an open-source MATLAB GUI called DL-QC-fNIRS. This tool provides researchers with a code-free environment that is accessible and offers robust channel-wise quality control.

**Fig. 4:**
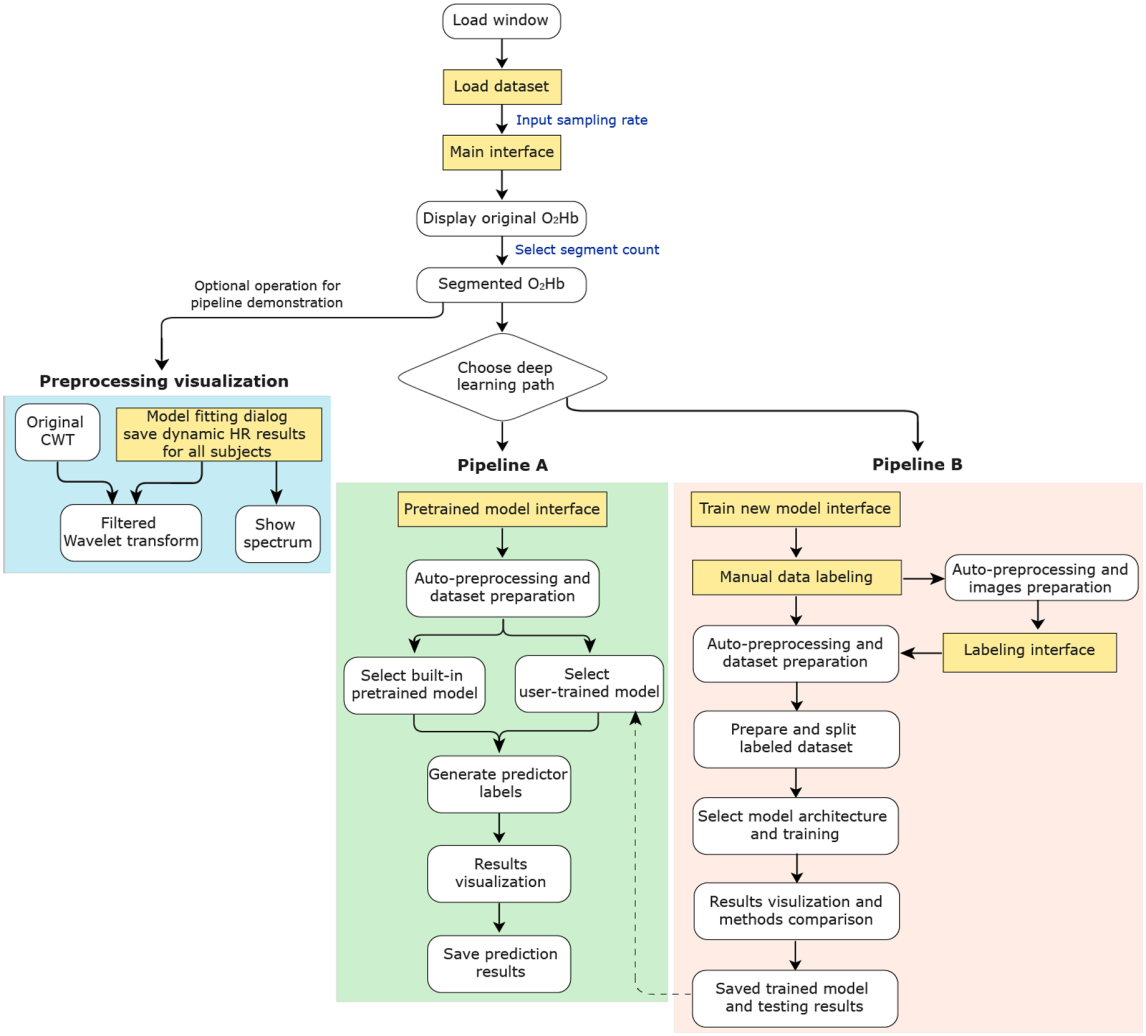
Operational flowchart of the DL-QC-fNIRS tool. The workflow illustrates the two main operational pipelines: Pipeline A streamlined assessment using a pretrained model for rapid analysis, and Pipeline B, a comprehensive framework for training a new model with a custom dataset, which includes modules for data annotation, model training, and validation.

The GUI supports both single-subject and batch processing, enabling users to either step through each preprocessing stage interactively or execute the entire workflow automatically with a single click. Pretrained DL models are included for immediate application, and a built-in annotation tool and predefined architectures enable users to train and evaluate custom models using their own datasets. The interface also provides real-time visualization of intermediate outputs and automatically stores the final quality assessment results (Fig. 4).

### 4.2 Loading Data and Preprocessing Modules

The GUI supports the import of data in the .snirf and .nirs format, ensuring compatibility with the widely used Homer2/Homer3 software suite, as well as in a structured .mat format. The .mat file must contain a structured array with the following mandatory fields: optical density data (d) and probe geometry (SD). There are also optional fields for the time vector (t), event markers (s), auxiliary channels (aux), and subject age (age). During data loading, users specify the signal sampling rate.

**Fig. 5:**
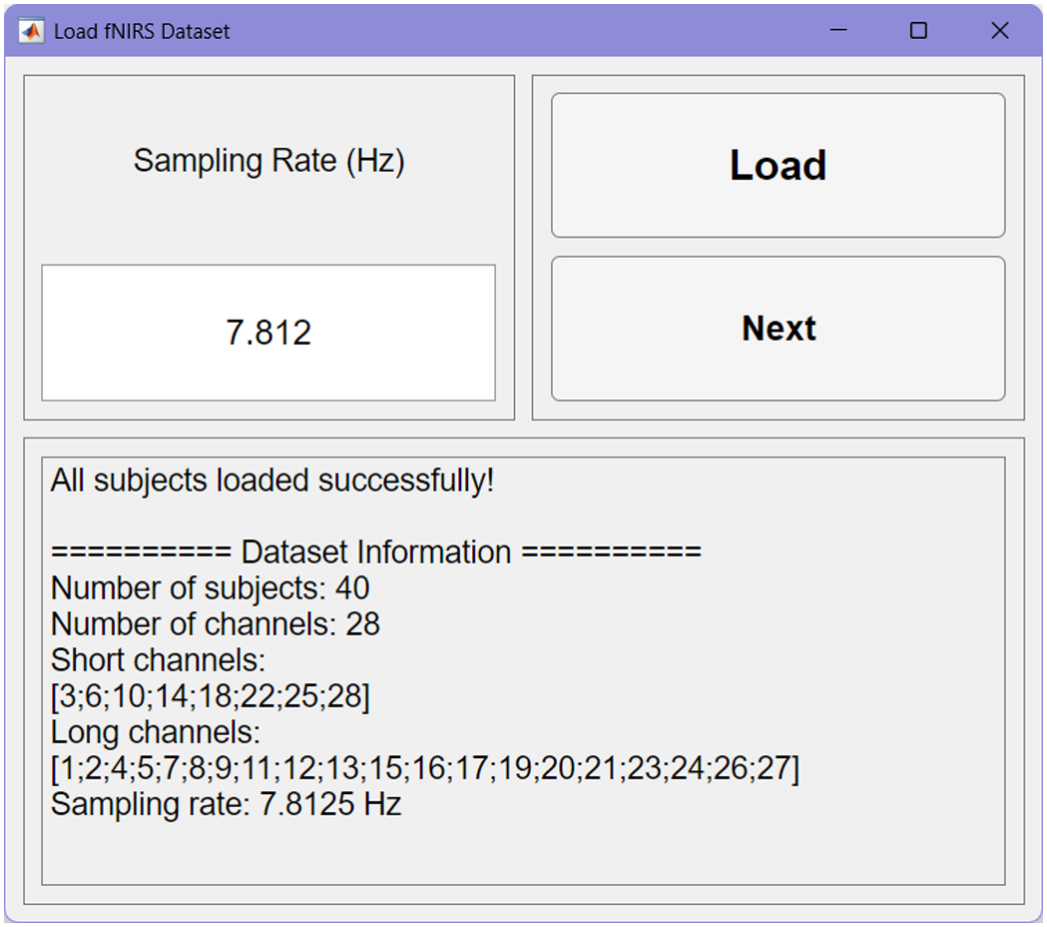
Data loading interface. The panel allows users to select datasets in .snirf, .nirs, or .mat format and supports batch processing of multiple files. Users must input the sampling rate and can optionally provide subject age for age-dependent DPF calculation.

To improve the accuracy of hemoglobin concentration conversion, the software implements an age-dependent differential pathlength factor (DPF) calculation.^77^ When subject age is provided, this correction mitigates the bias introduced by applying a fixed DPF value. The interface also allows entire datasets to be loaded in batches, enabling the efficient preprocessing of large studies (Fig. 5).

**Fig. 6:**
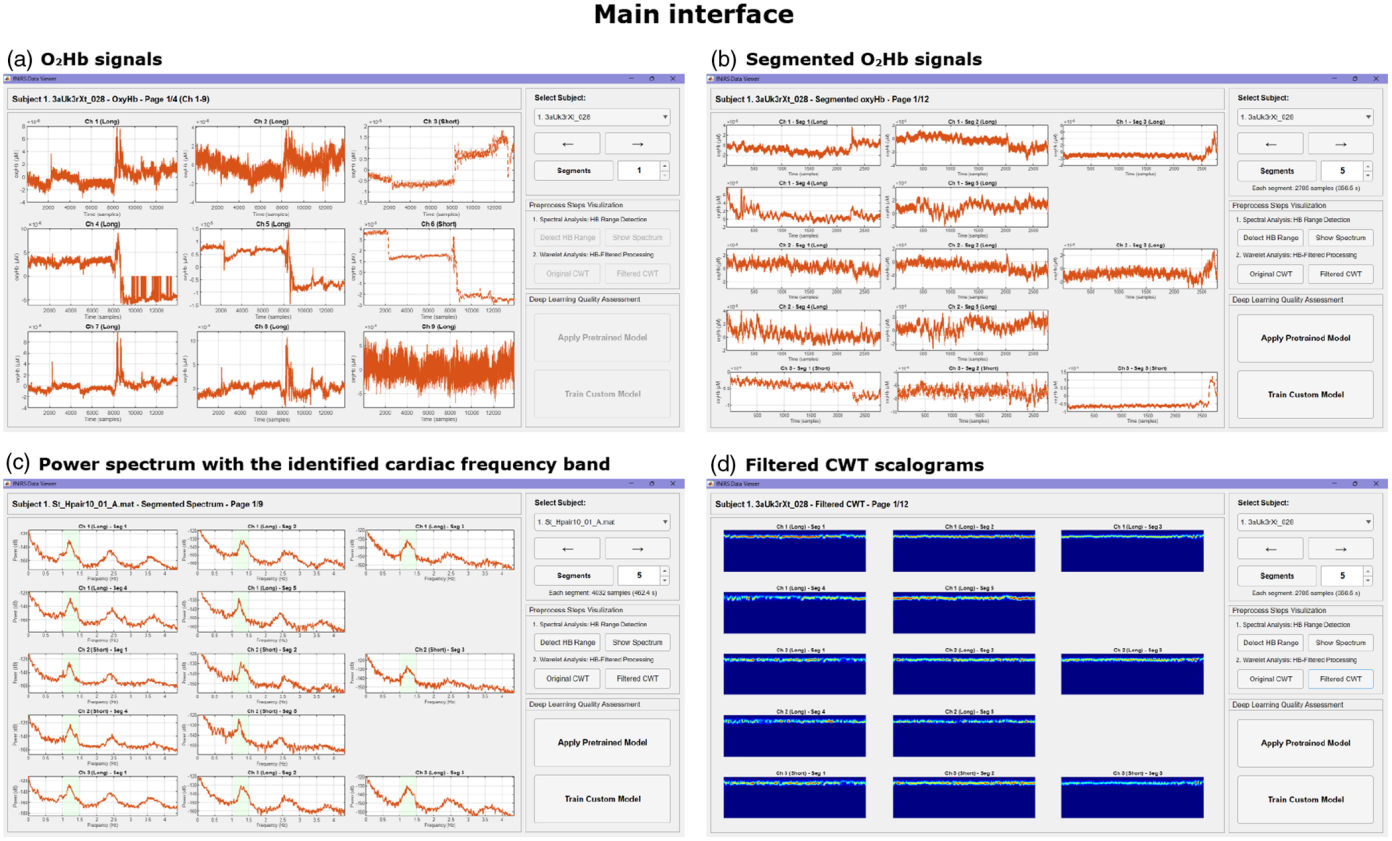
Preprocessing visualization in the DL-QC-fNIRS interface. (a) The interface displays the continuous oxyhemoglobin ($O_{2}\mathrm{Hb}$) signals for all channels of the selected subject. (b) The signal is then segmented into fixed-length windows chosen by the user. (c) The power spectrum of each segment is computed, with the individualized cardiac frequency band highlighted. (d) The corresponding continuous wavelet transform (CWT) scalogram is shown after band-specific filtering and normalization, ready for input to the neural network.

Once the data have been successfully imported, the main interface will display the oxyhemoglobin signals for all channels of the selected subject. Using the GUI, the user can specify the number of temporal segments into which each channel will be divided for quality assessment. After segment definition, users can either step through the preprocessing pipeline interactively or proceed directly to the DL module. The visualization workflow begins with spectral analysis, in which the optimal cardiac frequency band specific to the subject is identified. At this stage, the results of spectral fitting for all subjects in the dataset can be stored. Users can then inspect the estimated power spectrum with the cardiac band highlighted, the raw CWT scalogram, and the final, individualized CWT scalogram restricted to the cardiac band (see Fig. 6).

### 4.3 Deep Learning Module

The DL module offers two complementary pipelines to provide for different user requirements: one for rapid evaluation using pretrained models and another for training custom models using new datasets.

**Fig. 7:**
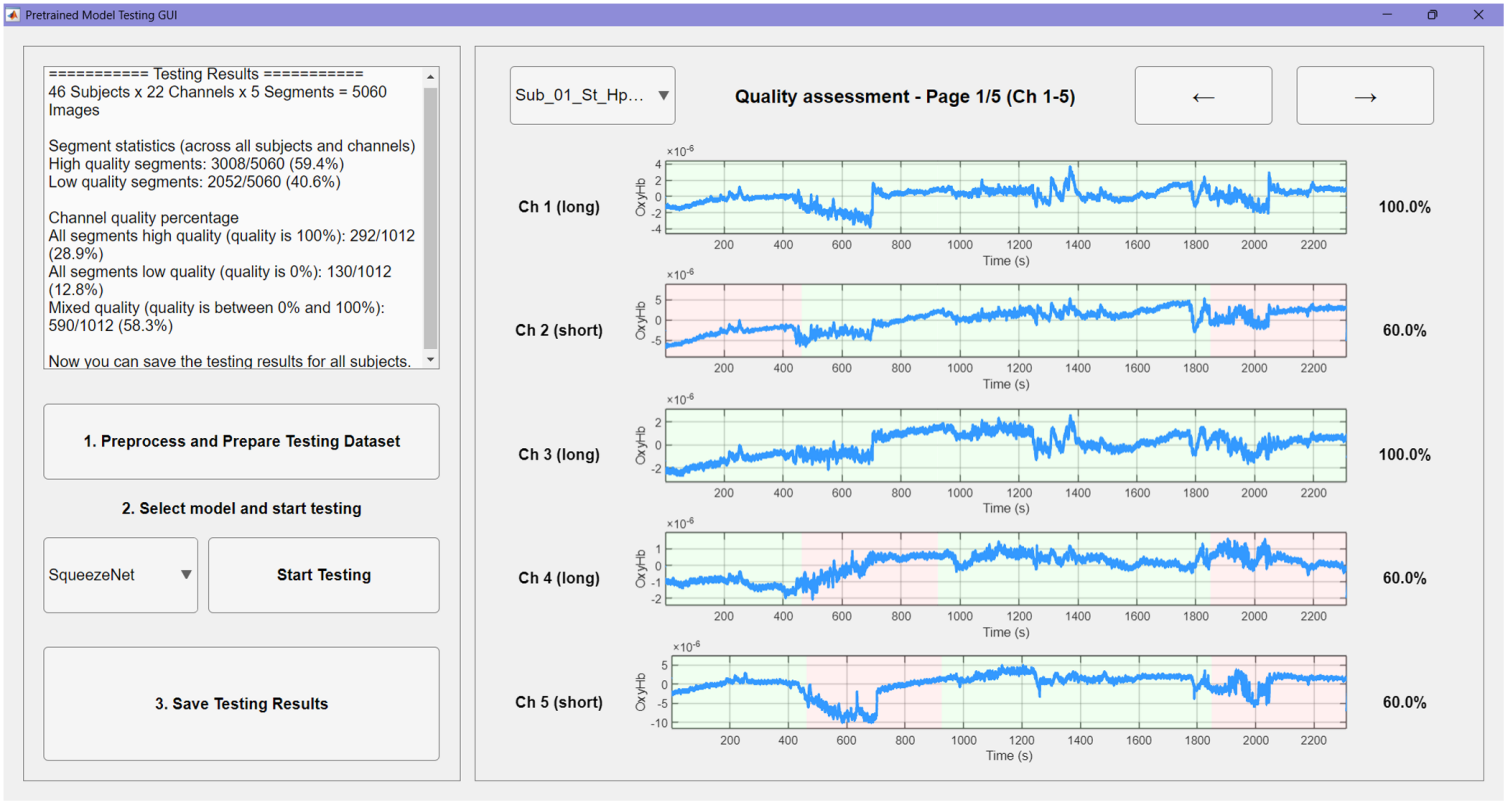
Pretrained model interface for automated signal quality evaluation. Each channel from the selected dataset is divided into five-time segments, which are individually classified using the pretrained model. Segment quality is color-coded: green indicates high-quality and red indicates low-quality. On the right, a summary panel displays the quality percentage for each channel, calculated as the proportion of segments classified as high-quality.

In the “Apply pretrained model” pipeline, users can select one of four architectures that have been pretrained on our dataset (SqueezeNet, GoogLeNet, ResNet, or EfficientNet) or load models that have been previously trained using the software. Once initiated, the pipeline automatically preprocesses the raw input and generates the adaptively filtered and normalized CWT scalograms required for classification. The GUI then visualizes the results and reports both segment-level quality predictions based on the user-defined segmentation (red for low quality, green for high quality) and aggregated quality percentages for each channel (see Fig. 7).

**Fig. 8:**
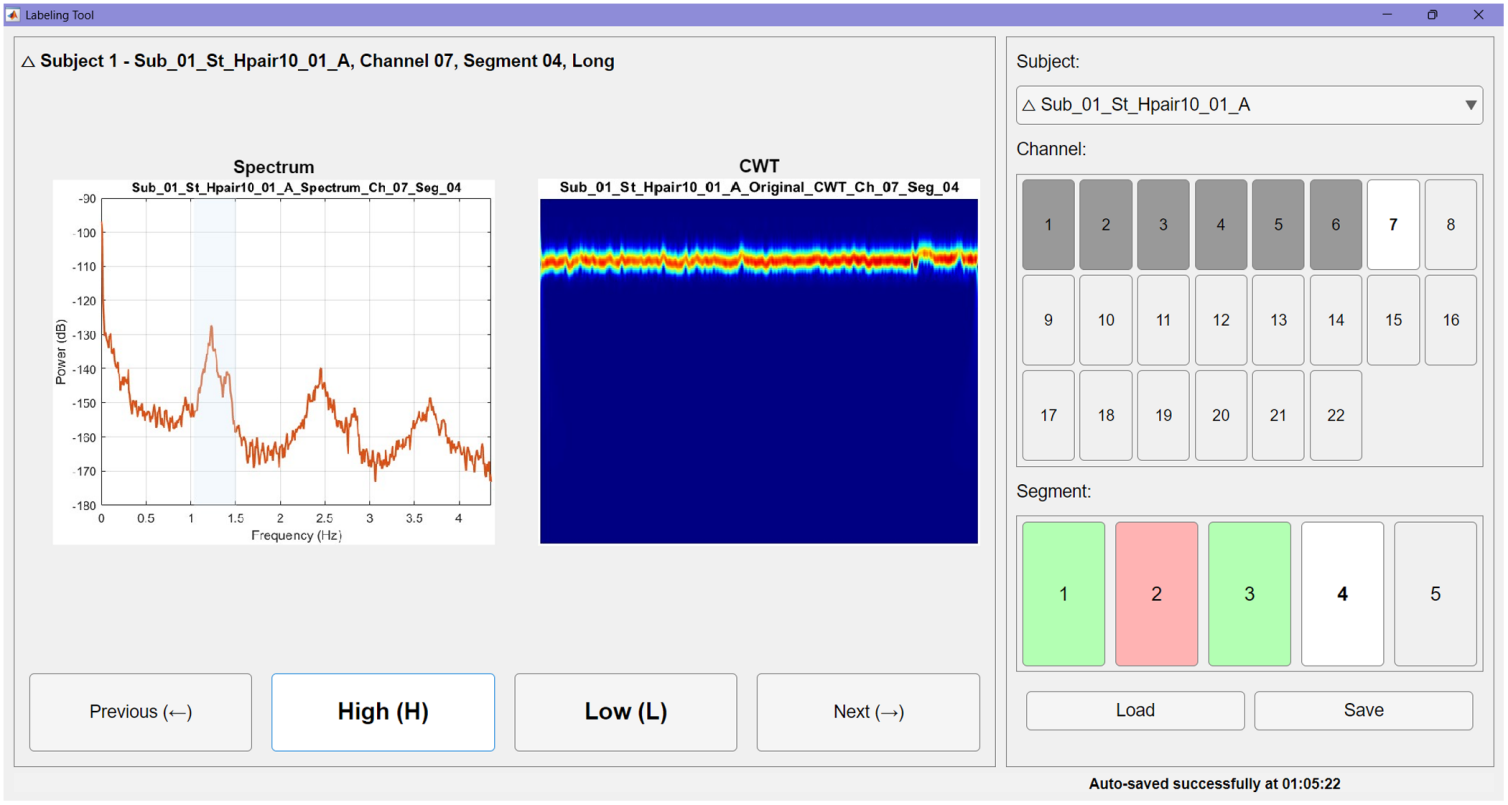
fNIRS visual labeling tool within the “Train Custom Model” pipeline. The interface supports manual annotation of signal segments by displaying both spectral and CWT scalogram representations. The control panel (right) allows users to navigate across subjects, channels, and segments. Channel buttons are color-coded: gray indicates incomplete annotation, and dark gray indicates completion. Segment buttons use red to mark low-quality (low) segments and green for high-quality (high). Annotation progress is tracked at multiple levels, and autosave/session recovery features ensure continuity during labeling.

The “Train Custom Model” pipeline enables users to build specialized models tailored to their own datasets. An integrated fNIRS visual labelling tool streamlines manual annotation by displaying spectral and CWT scalogram representations of each segment, enabling efficient labelling of segments as either high-quality or low-quality. The interface tracks progress at the subject, channel, and segment levels, while autosave and session recovery ensure continuity (see Fig. 8).

**Fig. 9:**
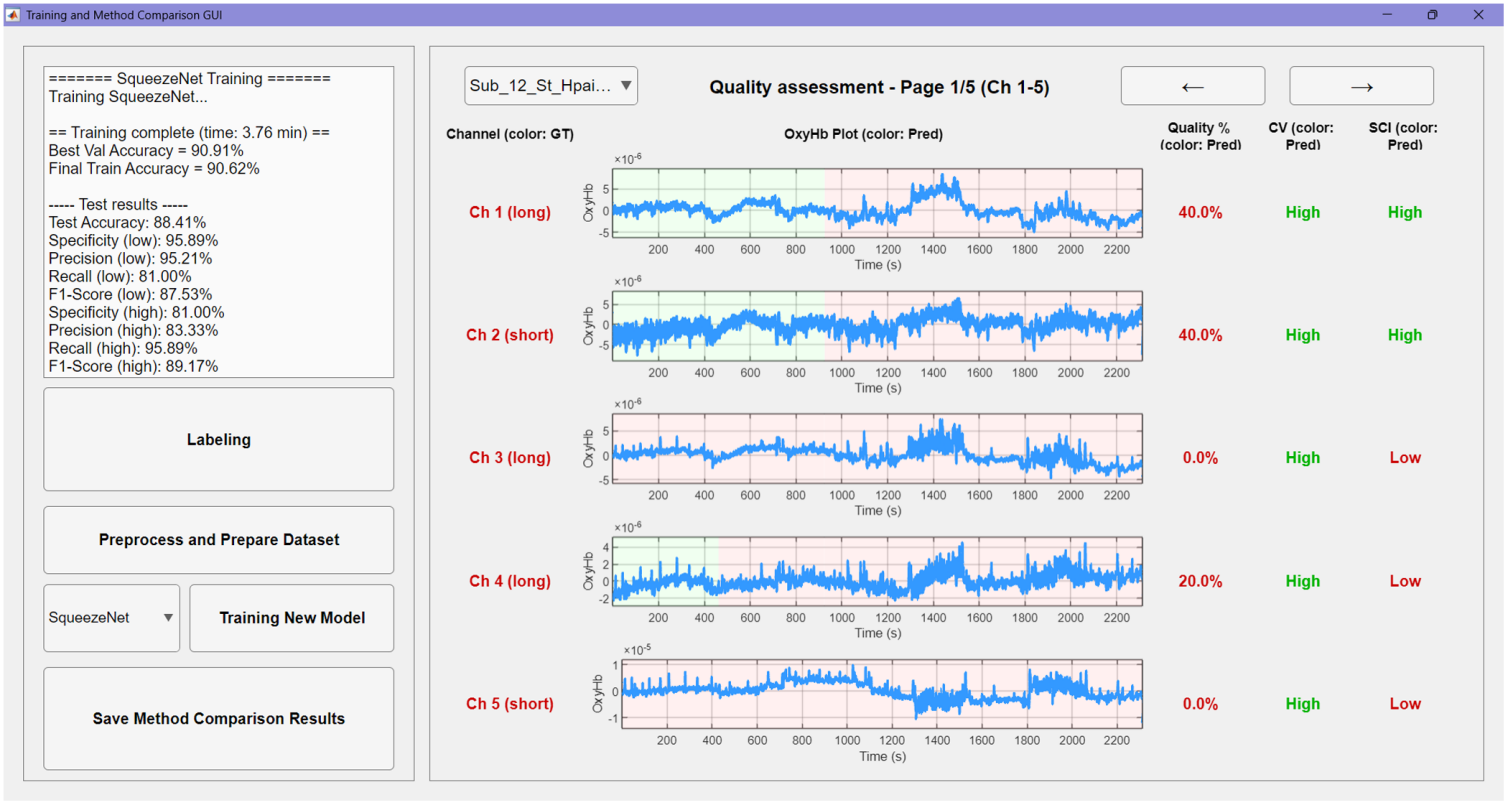
Interface for evaluating performance in the “Train Custom Model” pipeline. Once training is complete, the interface displays test set results for the selected model. The left panel shows the ground truth quality status of each channel, color-coded by the proportion of low-quality (low, red) and high-quality (high, green) segments. The center panel visualizes the segment-level predictions of the DL model. The right panel provides a side-by-side comparison of quality percentages computed by the trained model, the coefficient of variation (CV, threshold > 15%), and the scalp coupling index (SCI, threshold <0.75). Performance reports and trained models can be saved and reused in the “Apply Pretrained Model” pipeline.

Once annotation is complete, the labeled dataset is automatically partitioned into training, validation, and test sets in an 80:10:10 ratio. After selecting a model architecture and completing training, the user can generate a detailed evaluation report on the test set (see Fig. 9). To benchmark performance, the interface provides a direct side-by-side comparison between the DL model and two established quality metrics: the CV, applied with the commonly used 15% threshold, and the SCI, evaluated with the 0.75 threshold recommended by the original authors. Newly trained models, together with their associated performance reports, can be saved and subsequently reloaded into the “Apply pretrained model” pipeline for use with other datasets.

## 5 Discussion

### 5.1 Comparative Performance with Traditional Metrics

The results of our study demonstrate that our DL framework offers a more effective approach to fNIRS channel quality control than traditional, index-based metrics. Across both independent datasets and their combination, DL-QC-fNIRS (using GoogLeNet) achieved an accuracy of approximately 90% or above while maintaining strong sensitivity and specificity in detecting poor-quality channels. By contrast, the CV method and the SCI exhibited highly skewed sensitivity–specificity performance. For instance, the CV threshold (set to 15% in this study) was overly conservative, achieving near-perfect specificity (∼99%), yet exhibiting extremely low recall (less than 25% in one dataset). This aligns with prior observations that, although popular, a single high CV cutoff lacks sensitivity to many subtle noise issues and may only detect extreme signal fluctuations.^4^^,^^67^ Similarly, the SCI with a fixed threshold of 0.75 achieved high recall (often >90%), but this came at the cost of extremely low specificity (as low as ∼48% in our results). These outcomes highlight the limitations of index-based signal quality indices, which exhibit either very low recall or very low specificity, depending on how the threshold is set.

Our DL-based approach effectively overcomes these issues by identifying the complex, multifactorial patterns of high-quality versus low-quality channels rather than relying on a single heuristic cutoff. Notably, it achieved the high recall that CV lacked while also maintaining the high specificity that the SCI failed to provide. This resulted in the highest F1 scores in all comparisons.

### 5.2 Alignment with Recent Literature

These findings are in line with emerging trends in fNIRS quality control research. Earlier work by Pollonini et al. and Sappia et al. introduced quantitative scalp-coupling and signal-quality indices that provided an important foundation for automated quality assessment.^12^^,^^13^ However, as summarized in recent reviews by Klein, such index-based approaches remain limited by their reliance on heuristic thresholds and their inability to generalize across subjects and devices.^5^

Bizzego et al. demonstrated that, in principle, conventional signal quality indicators (SQIs) may distinguish poor-quality signals, but applying fixed, nonadaptive thresholds to these metrics often results in many poor-quality channels being missed, thereby reducing sensitivity.^78^ Machine learning (ML) approaches have been shown to offer a solution by combining multiple features or using data-driven classification to achieve greater accuracy than any single metric alone. Indeed, the same researchers reported that, although individual SQIs were statistically different for low-quality versus high-quality signals, index-based filtering lacked sensitivity, and classification models (including convolutional neural networks) significantly improved detection performance.

More recently, Gerloff et al. introduced NiReject, a set of ML detectors (including unsupervised and semi-supervised models) for fNIRS signal quality control.^10^ Their comprehensive evaluation on ∼30,000 signals showed that data-driven models outperformed traditional threshold methods, especially when some level of training data was available. Notably, they found that inadequate low-quality channel detection may strongly bias neuroimaging results, yet only ∼32% of published fNIRS studies explicitly report employing any low-quality channel identification technique. This underlines the critical need for robust, automated quality check tools.

Together with recent studies highlighting the role of physiological and anatomical variability in determining signal quality (Yücel et al.), these results underscore a clear shift toward data-driven, standardized frameworks for fNIRS quality control.^79^ Our work contributes to this movement by providing a fully supervised deep-learning solution that is both accurate and practical, complementing existing semi-supervised and feature-based approaches toward reliable fNIRS data quality assurance.

### 5.3 Deep Features and Time-Frequency Analysis

The strong performance of our DL-QC-fNIRS framework was likely due to several factors. Firstly, using an individualized cardiac frequency band for each subject enhanced the fidelity of physiological feature extraction. Automatically identifying the heart-rate peak of each subject in the power spectrum enabled us to isolate the cardiac pulsation signal with greater precision than would have been possible using a broad fixed band. Consequently, the CWT scalograms fed to the network contained a clear representation of the heartbeat oscillation (or its absence) for each channel. The presence of stable cardiac pulsations throughout the recording indicates good optode contact and data quality, whereas a reduction or disappearance often signifies decoupling or saturation artifacts. Traditional metrics that use a generic cardiac band may either miss the pulsation if the heart rate is outside the assumed range or include noise frequencies that confuse the metric. Our adaptive band approach mitigates these issues and was instrumental in enabling the CNN to learn more reliable signal quality features. This level of personalization improves sensitivity to true positives (the detection of subtle but real cardiac signals in high-quality channels) while avoiding false positives caused by off-band noise. It should be noted that the concept of adapting to individual cardiac frequencies is not unique to our approach; similar strategies have been adopted in tools such as Phoebe (Pollonini et al.) to refine scalp–coupling estimation.^13^ However, in DL-QC-fNIRS, this individualization is integrated into a deep-learning framework, enabling the model to learn complex, multidimensional representations of signal quality rather than relying on explicitly defined indices.

Another strength of our method is the time-frequency analysis offered by wavelet scalograms. Rather than reducing each channel to a single summary metric, the CNN examines the entire recording segment in the joint time–frequency domain. This offers two advantages. (i) Temporal dynamics: the model is able to detect transient changes in quality. For instance, if a channel initially performs well but subsequently loses the heartbeat signal (possibly due to probe slippage), our approach is able to identify this deterioration, whereas a static metric averaged over time might dilute or overlook it. Conversely, brief motion artifacts that momentarily disrupt the signal appear as transient bursts of broadband power in the scalogram, and the CNN learns to discount such short-lived events if the channel is otherwise high-quality. Essentially, the decision of the network reflects the proportion and timing of high-quality versus low-quality periods within a segment, much like an expert human reviewer watching a time-course movie of the data. In practice, because the framework operates at the segment level, DL-QC-fNIRS can also identify temporal variations in channel quality across consecutive subsegments. For instance, if a brief movement causes temporary coupling loss, the affected subsegment will be marked as low-quality, while subsequent segments showing restored cardiac pulsations will be classified as high-quality. This segment-based design enables detection of postmovement coupling loss and recovery without relying on past data, providing users with a time-resolved view of signal quality. The final decision on whether to exclude a specific subsegment or discard the entire channel can be made based on the percentage of low-quality segments displayed in the GUI. Because the recordings analyzed in this study were acquired during baseline resting-state conditions, longer (6 to 7 min) segments were intentionally used to capture overall signal stability rather than rapid temporal fluctuations. Nevertheless, the framework and software are flexible and can operate on shorter or sliding windows for applications requiring higher temporal resolution, such as real-time artifact detection. (ii) Multifeature integration: the CWT representation inherently captures multiple facets of signal quality. The strength and continuity of cardiac oscillations, the level of noise across various frequencies (from slow drifts to high-frequency sensor noise), and sudden anomalies (such as spikes or step changes) all appear as distinguishable patterns in the scalogram. Our CNN has effectively learned to interpret these patterns holistically. Unlike composite indices, which manually combine a few metrics (e.g., combining CV, SCI, and other measures with fixed weights, as described by Sappia et al.),^12^ our deep network automatically learns to integrate multiple quality-related features in a nonlinear fashion. Although our empirical comparison was limited to single-feature metrics (CV and SCI), this capacity for learned feature fusion conceptually explains the superior accuracy and F1-scores achieved by DL-QC-fNIRS.

### 5.4 Model Selection and Generalization Across Datasets

It is also noteworthy that the GoogLeNet architecture emerged as the top performer in the tests. In cross-validation, GoogLeNet slightly outperformed both a very deep model (ResNet-50) and a very compact model (SqueezeNet), suggesting that it strikes an effective trade-off between complexity and generalization. The fact that SqueezeNet and EfficientNet-B0 achieved 89% to 93% accuracy in cross-validation indicates that the scalogram information is robust and learnable, even with smaller models or fewer parameters. This is encouraging for real-world deployment, as a lighter model could be used for real-time applications or on less powerful hardware with only a minor trade-off in accuracy if needed. To quantify this trade-off, we benchmarked the total inference time per segment (including an ∼200  ms preprocessing overhead) and found it to be ∼201  ms for SqueezeNet, 202 ms for GoogLeNet, 204 ms for ResNet-50, and 209 ms for EfficientNet-B0. We also found that rule training on the merged dataset produced the best results (over 93% cross-validation accuracy), highlighting the importance of diverse training data. By exposing the model to two different instruments, montages, and subject populations, we effectively regularized it to focus on general quality-related features rather than device-specific idiosyncrasies. The high test accuracy (∼92%) on the combined dataset’s hold-out subjects demonstrates the generalizability of the model. Although the present model was trained on 6 to 7 min segments, its performance does not depend on absolute window length but on the presence of stable cardiac-band oscillations. Consequently, DL-QC-fNIRS can also be applied to shorter windows, provided that they include several cardiac cycles (≈10 to 15 s). Because the CNN operates on normalized and rescaled wavelet scalograms (224×224  pixels), its performance is largely independent of the original sampling rate. Future work will extend validation to higher-frequency data and active tasks to further assess model generalization. In practical terms, this means that our approach has demonstrated generalization across datasets acquired with similar hardware configurations. Future studies will be necessary to assess performance across fNIRS systems from different manufacturers and with a broader range of sampling rates. As more varied datasets become available, they may be used to retrain or fine-tune the model (including those involving different age groups, pathologies, motion conditions, etc.), which may further enhance its versatility and robustness.

Although DL-QC-fNIRS applies a binary classification at the segment level (“high” versus “low” quality), this design was intentionally chosen to reflect the practical accept/reject decisions required in standard fNIRS preprocessing. As reported by Yücel et al., hair and skin characteristics substantially influence fNIRS signal quality, producing intermediate cases that fall between clearly good and poor channels.^79^ To accommodate this gradual variation, the software provides a channel-level quality percentage indicating the proportion of high-quality data, enabling the users to define flexible acceptance thresholds rather than relying on a strict binary decision.

### 5.5 Practical Implications and Software Integration

From a user perspective, one of the most significant contributions of this work is the integration of the DL pipeline into an open-source MATLAB GUI tool (see Sec. 4). Advanced signal processing or ML methods are often out of reach for nonexpert users, hindering their impact on day-to-day research practices. By providing a plug-and-play interface with pretrained models, this work aims to bridge the gap between sophisticated algorithms and laboratory workflows. Researchers can now perform rigorous, channel-wise quality assessments without writing a single line of code or understanding the intricacies of wavelet transforms or neural networks. The GUI simplifies the entire process, from loading data and performing automatic spectral analysis and scalogram generation to producing final quality reports, and offers transparency. The users can visualize the spectra, the identified cardiac band, and the scalograms and manually adjust or label data if necessary.

### 5.6 Limitations

Despite the promising results, our study has several limitations that warrant discussion. First, both the training and evaluation datasets consisted of adult resting-state recordings, with subjects at rest aside from natural, unconstrained motion. It remains to be seen how the model will perform with data from active tasks or different populations (e.g., children or clinical groups), where signal characteristics may differ. Task-evoked hemodynamic changes could introduce nonstationary components that the model has never encountered before (because our high-quality segments mostly consisted of a relatively flat baseline with only physiological oscillations). In such scenarios, the network might mistake a large stimulus-evoked surge in $\left[O_{2}\mathrm{Hb}\right]$ for an artifact if it falls outside its learned patterns. However, because we deliberately focused on cardiac-frequency features and general noise patterns, we are optimistic that the model will primarily identify low-quality channels by their absence of physiological rhythms rather than their presence of neural responses. Nevertheless, future work should validate the model on task-based data and incorporate examples of both baseline and activation periods in the training process to ensure robustness in these conditions. We also note that some types of motion artifacts may temporarily reduce or obscure the cardiac pulsation even when optode–scalp coupling remains adequate. In such cases, DL-QC-fNIRS, similar to other index-based quality measures, may classify these segments as low-quality because the physiological rhythm is not visible. Future evaluations on datasets with more diverse and controlled movement patterns will help clarify and refine the method in such situations.

Another limitation is that, as with any supervised learning approach, the performance of our model is tied to the quality of the training labels. Although we took care to ensure annotation of the segments and employed a second dataset to increase heterogeneity, there is always a possibility of label noise or systematic bias in the reference labels. What one expert considers low-quality (especially if based on subtle noise levels), another might tolerate. Our model will inherit these definitions. In the future, gathering annotations from multiple experts and using consensus or adjudication could improve label quality further. Techniques such as semi-supervised learning or active learning could also reduce the need for exhaustive labeling by enabling the model to identify cases that require expert review.

In addition, model interpretability poses a challenge. Deep CNNs are often criticized for being “black boxes”. Although our network clearly identified physiologically sensible features (for example, it distinguishes between channels with and without cardiac pulsations), we did not explicitly visualize the learned filters or employ explainable artificial intelligence techniques in this study. Using methods such as class activation mapping or occlusion analysis could reveal which aspects of the scalogram most influence the decisions of the network. This would validate that the model is focusing on meaningful signal attributes and not on artifacts of the colormap or edge effects, for example, and could potentially uncover new patterns indicative of signal quality that we did not consider.

Another methodological consideration is that our comparison with traditional metrics (CV and SCI) followed the most common threshold settings reported in the literature. These established metrics were selected as widely adopted baselines to ensure reproducibility. However, other recent methods could perform differently under optimized or alternative parameter choices. Future studies should therefore include comparisons across multiple index-based and ML/DL approaches.

Lastly, we acknowledge that our method currently focuses on $\left[O_{2}\mathrm{Hb}\right]$ signals and uses a segment length (several minutes) that was convenient for our resting-state data. Different analytical requirements may necessitate adjustments. In particular, the current implementation was developed and validated offline using long segments; however, the underlying architecture is compatible with real-time operation based on short sliding windows, which will be explored in future work. For instance, one might wish to evaluate quality in shorter time frames (to enable near-real-time feedback) or incorporate concentration changes in deoxyhemoglobin ([HHb]) signals to detect issues such as wavelength-specific noise. In addition, multiwavelength decomposition approaches, including the orthogonal-component analysis of Umeyama and Yamada and the characterization of nonhemodynamic signal components in continuous-wave fNIRS by Yamada, provide a complementary perspective on optode–scalp contact.^80^^,^^81^ In these frameworks, the component of the absorbance signal orthogonal to the $\left[O_{2}\mathrm{Hb}]/[\mathrm{HHb}\right]$ extinction subspace captures nonhemodynamic variations and can therefore serve as an additional indicator of optode–scalp coupling. Incorporating such physically motivated components or combining $\left[O_{2}\mathrm{Hb}\right]$- and [HHb]-based scalograms represents a promising direction for future development of DL-QC-fNIRS, as a truly low-quality channel would be expected to show degradation across both wavelengths, whereas systemic artifacts may affect them differently.

Another interesting possibility for future work would be to extend the model to include multiple input channels ($\left[O_{2}\mathrm{Hb}\right]$ and [HHb] scalograms together), as a truly low-quality channel would presumably show issues in both, whereas a systemic artifact might affect them differently.

## 6 Conclusion

This study introduces DL-QC-fNIRS, a DL-based framework for the automated assessment of fNIRS channel quality. By combining time–frequency representations with an ML/DL-based model, the approach effectively identifies poor-quality channels without relying on heuristic thresholding. Evaluated across two independent datasets and a combined heterogeneous dataset, DL-QC-fNIRS consistently outperformed traditional index-based metrics such as the CV and the scalp coupling index SCI, achieving higher accuracy and F1-scores.

The framework is implemented in an open-source, MATLAB-based GUI, enabling researchers to perform standardized quality control without coding expertise. This accessibility supports broader adoption and promotes more rigorous, reproducible fNIRS research.

Future work will extend validation to diverse populations, experimental tasks, and real-time applications. Nonetheless, our results demonstrate that DL serves as a virtual expert for fNIRS signal quality, enabling scalable and reliable automation of data quality assurance.

## Data Availability

All code and data associated with this study are publicly available in our GitHub repository: https://github.com/zhuofeichen312/DL-QC-fNIRS. This repository includes the source code for the software and GUI, the full scripts for model training, our final pretrained models, and the datasets used in this study.
